# Factors influencing possible delay in the diagnosis of Alzheimer's disease: Findings from a tertiary Public University Hospital

**DOI:** 10.1590/S1980-57642011DN05040011

**Published:** 2011

**Authors:** Luís Felipe José Ravic de Miranda, Rafael de Oliveira Matoso, Márlon Vieira Rodrigues, Thiago Oliveira Lemos de Lima, Adriano Fiorini Nascimento, Fernando Castro Carvalho, Débora Regina de Melo Moreira, Jeferson Cruz Fernandes, Jonas Jardim de Paula, Luiz Alexandre V. Magno, Paulo Caramelli, Edgar Nunes de Moraes

**Affiliations:** 1 Department of Clinical Medicine, Federal University of Minas Gerais, Belo Horizonte MG, Brazil; 2 School of Psychology, Federal University of Minas Gerais, Belo Horizonte MG, Brazil; 3 Research Laboratory of Neuropsychology of the Federal University of Minas Gerais, Belo Horizonte MG, Brazil; 4 Molecular Medicine - Neuroscience Laboratory, INCT of Molecular Medicine, Federal University of Minas Gerais, Belo Horizonte MG, Brazil; 5 Department of Clinical Medicine, Federal University of Minas Gerais, Belo Horizonte MG, Brazil

**Keywords:** Alzheimer's disease, delay in diagnosis, education, caregivers

## Abstract

Alzheimer's disease (AD) is characterized by impairment in memory and autonomy, causing excessive pressure on family and an overburdened health care system. Early diagnosis, with the appropriate treatment, is important to reduce the pattern of disease progression.

**Objective:**

The study sought to identify the most probable causes of delay in diagnosis.

**Methods:**

A cross-sectional study involving AD patients followed at an Outpatient Geriatric Clinic from a tertiary public university hospital was conducted between June 2009 and February 2011.

**Results:**

Ninety-four patients were evaluated (66% women), aged 77.76±6.8 years and with median educational level of 3 years (95% CI 2.7-3.80). Regarding severity of dementia, 51.8% of patients were classified as having mild dementia (CDR 1), 40% moderate dementia (CDR 2) and 8.2% severe dementia (CDR 3). Mean educational level of caregivers was 8.3±3.9 years. Among those who believed there was a delay, 36% stated that the "family thought that the changes were normal for the age of the patient" reporting average delay of 1.8 years (95% CI: 1.3-2.5) while 45.3% stated that the "doctor did not reach a diagnosis" reporting a median delay of 1.5 years (95% CI: 1.4-2.3).

**Conclusion:**

Based on these results, it can be concluded the time between onset of symptoms and diagnosis was excessive. This study may be useful to help increase awareness of issues not sufficiently discussed in the literature, such as diagnostic delay and influence of caregivers' educational level on treatment.

## Abstract (pt)

A doença de Alzheimer é caracterizada por comprometimento na memória e na autonomia, causando pressão excessiva em familiares e sobrecarregando o sistema público de saúde. O diagnóstico precoce, com o tratamento adequado, é importante para reduzir o padrão de evolução da doença.

**Objetivo:**

O estudo pretende identificar as causas mais prováveis de atraso no diagnóstico.

**Métodos:**

Trata-se de um estudo transversal envolvendo pacientes com DA acompanhados em Ambulatório de Geriatria de um hospital terciário público entre junho de 2009 e fevereiro de 2011.

**Resultados:**

Noventa e quatro pacientes foram avaliados (66% mulheres), com média de idade de 77,8±6,8 anos e com mediana de escolaridade de 3 anos (IC 95%: 2,7-3,8). Quanto à gravidade da doença, 51,8% foram classificados como demência leve (CDR 1), 40% como demência moderada (CDR 2) e 8,2% como demência grave (CDR 3). A escolaridade do cuidador foi de 8,3±3,9 anos. Entre aqueles que acreditavam que havia um atraso no diagnóstico, 36% responderam que "a família achava as alterações como normais para a idade do paciente", com média de 1,8 anos (IC 95%: 1,3-2,5) e 45,3% responderam que "o médico não fez o diagnóstico", com mediana de 1,5 anos (IC 95%: 1,4-2,3). Foi observado que o tempo entre o início dos sintomas e o diagnóstico foi maior do que deveria ser.

**Conclusão:**

Este estudo pode contribuir para aumentar o conhecimento sobre questões ainda pouco discutidas na literatura científica, como atraso no diagnóstico e influência da escolaridade do cuidador no tratamento.

## Introduction

Following the worldwide trend, Brazil has experienced a significant increase in life expectancy, rising from 62.6 years in 1980 to 73.1 years in 2009.^1^ As a result of this aging of the population, the prevalence of dementia is increasing and becoming a major public health problem. The prevalence of dementia in the United States is about 13%^2^ in individuals aged 65 years or older and, in Latin America, this figure stands at around 7.1%.^3^ In India, dementia is the second most common neuropsychiatric disorder.^4^

Dementia is characterized by many years of impairment, loss of autonomy and sometimes loss of independence, leading to family burden, loss of work activity and pressure on the health care system. In 2008, the cost for the Brazilian public health system (SUS) of distributing anticholinesterase drugs was approximately 100 million Reais, covering approximately 12% of all AD patients.^5^ Among the medications currently available for treatment, rivastigmine was the most prescribed (71.4%), followed by donepezil (26.2%) and galantamine (2.4%).^5^

The earlier the diagnosis is reached, the more the family and the patient benefit from the use of appropriate medication. Therefore, early intervention is extremely important for reducing the pattern of clinical course besides leading to symptomatic benefits.^6^ However, in clinical practice, time elapsed between the onset of symptoms and confirmation of diagnosis is longer than it should be while only a few studies highlight the causes behind this diagnostic delay.

The aim of this study was to verify the effects of different variables on diagnostic delay in a sample of AD patients followed at an outpatient clinic from a tertiary public university hospital.

## Methods

### Study design

This cross-sectional study was conducted at the Geriatric Outpatient Clinic of the Hospital das Clínicas at the Universidade Federal de Minas Gerais (UFMG), in Belo Horizonte (MG), Brazil.

### Population

The sample comprised patients evaluated between June 2009 and February 2011 at the Geriatric Outpatient unit of the Hospital das Clínicas of UFMG, who were at least 60 years of age, and had received the diagnosis of probable AD according to NINCDS-ADRDA criteria^7^ or of AD with cerebrovascular disease, according to NINDS-AIREN criteria.^8^ Only one patient was living in an institution for a long period of time, while all others lived with their families.

### Instrument and data collection

The patients' relatives answered questions as listed below and other information was collected by examining medical records.

The variables studied were age, gender, the *Clinical Dementia Rating* (CDR), length of delay of diagnosis (in years) - defined by the time between the onset of symptoms of cognitive decline and the clinical diagnosis, number of doctors consulted due to cognitive decline prior to establishing a diagnosis of dementia, causes for diagnostic delay, educational level of the patient and of the caregiver.

Age and CDR scores represent the values obtained at time of diagnosis. The time when symptoms of cognitive decline manifested was obtained through clinical history (interview with the family caregiver).

Among the causes for diagnostic delay, the family members chose from among the following options: "thought it didn't take long", "thought the changes were normal", "the doctor did not reach a diagnosis", "family incapacity to provide care", and "none of the above".

### Statistical analysis

Data analysis was carried out using the *Statistical Package for the Social Sciences (SPSS) version 19* and took into consideration descriptive statistics. The Kolmogorov-Smirnov test was used to evaluate the distribution pattern of the variables, the Chi-Square tests to compare proportions, one-way ANOVA to compare means, and Kruskal-Wallis to compare medians, adopting a significance level of 5%. The variables with normal distribution are expressed as mean and standard deviation values, while the others are shown as median and interval of confidence (95%).

## Results

Ninety-four patients were evaluated (66% female), aged 77.8±6.8 years and with median educational level of 3 years (95% CI 2.7-3.8). Regarding severity of dementia, 51.8% of patients were classified as having mild dementia (CDR 1), 40% moderate dementia (CDR 2) and 8.2% as severe dementia (CDR 3) (Table 1).

**Table 1:** Profile of patients diagnosed with AD.

Gender (%)	Female	66
	Male	34
Mean age		77.8±6.8 years
Mean education level of caregiver		8.3±3.9 years

The age of the caregiver had a mean of 52.43±11.41 years and educational level had a mean of 8.29±3.90 years. In the sample studied, no statistical relationship was identified between educational level of caregivers and delay in diagnosis. This showed that caregivers with a higher level of education did not obtain an earlier diagnosis for their sick relatives.

The length of time between the onset of symptoms of cognitive decline and the diagnosis of dementia had a median of 1.5 years (95% CI 1.6-2.2).

Before obtaining a diagnosis, the median number of doctors sought by the patients was two (95% CI 1.7-2.1) (Table 2). Those who were diagnosed by the first doctor presented a median delay of 1.0 year (95% CI: 1.0-1.7), for two doctors the median was 1.8 years (95% CI: 1.6-2.6), for three doctors the median was 1.7 years (95% CI: 1.1-2.5) and those who consulted four physicians presented a median diagnostic delay of 3.2 years (95% CI: 0.1-5.6). There was a weak relationship between interval from onset of symptoms to confirmed diagnosis and number of doctors seen (R=0.27, p=0.014).

**Table 2:** Delay in diagnosis.

Median length of delay		1.5 years (95% CI: 1.6-2.2)
Median number of doctors		2 doctors (95% CI: 1.7-2.1)
Cause (%)	Considered changes normal	32.5
	Doctor did not diagnose	41.0
	Family incapacity to provide care	4.8
	None of the above	12.0
	Thought it did not take long	9.6

A significant relationship was observed between the time of symptoms and possible causes for the delay in diagnosis. Overall, 9.6% of the caregivers considered "it did not take long", with median time between symptoms and diagnosis of 0.7 years (95% CI: 0.3-1.2). Among those that thought there was a delay, 36.0% stated that the "family thought that the changes were normal for the age of the patient", with a median diagnostic delay of 1.8 years (95% CI: 1.3-2.5) while 45.3% stated that the "doctor did not reach a diagnosis", with a median of 1.5 years (95% CI: 1.4-2.3). There was a significant difference between diagnostic delay and family incapacity to provide care. Nevertheless, only four patients' family members reported being incapable of providing care.

There were no significant differences between men and women for age at diagnosis, number of doctors consulted, stage of the illness, and delay time of diagnosis.

## Discussion

The median number of doctors sought by patients in the present study was 2 (95% CI 1.7-2.1), while the median time between the onset of symptoms of cognitive decline and the diagnosis of dementia was 1.5 years (95% CI 1.6-2.2). No association was found between educational level of the caregivers and diagnostic delay. The main cause accounting for the diagnostic delay, from caregivers' viewpoint, was the inability of the physician to reach a conclusion.

These findings may be interpreted as follows: some doctors do not fully understand the diagnostic criteria of dementia and AD. The patients whose families were unable to provide adequate care had a much more delayed diagnosis. This can have an impact on public health since the family is not always familiar with the clinical features of the disease and treatment issues. Only four families reported family inability to provide adequate care, precluding the drawing of further conclusion. Another common reason for diagnostic delay was the belief that cognitive impairment in elderly people is normal.

In the national health care system (SUS), the patient is generally evaluated by a general practitioner (initial consultation) and later referred to a specialist - geriatrician, neurologist or psychiatrist. The number of doctors who were seen by our patients (median=2) is consistent with the Brazilian service delivery system. However, the number of doctors consulted is a possible factor contributing to delay in diagnosis since the time between the first consultation and the diagnosis of the disease is prolonged. Hence, patients not treated with cholinesterase inhibitors may present increased cognitive decline representing precious lost time for patients and their families.^9,10^

There was an inverse association between the number of doctors and educational level of the caregiver, i.e. higher educational level did not result in a lower number of doctors consulted. No previous studies were found reporting correlation between educational level of the caregiver and access to earlier diagnosis and treatment. The majority of the articles that refer to caregivers consider stress, symptoms of depression in response to situation faced, and the relief felt when the patient dies.^11^

Lay persons' understanding of the disease still seems limited. Concerted efforts have been made to address this problem at the public health level, such as provision of training to the team of professionals working in the public service network, and of information about the illness through collaborative service delivered to those suspected of having some degree of cognitive deficit.

The main limitation of this study is related to the small sample size evaluated. However, we believe that this study may call attention to some issues that are seldom discussed in literature, including possible delay in diagnosis, influence of caregivers' educational level on patient treatment, number of doctors the patient sees before a correct diagnosis is reached, and stage of illness on patient entry to the outpatient clinic to begin treatment.

The issues addressed in this paper need to be further investigated and included in the literature as they reflect the reality of AD patients in Brazil and the treatment they receive through the national health care system.
